# Case report: Side effects of etomidate in propylene glycol in five Göttingen Minipigs

**DOI:** 10.3389/fvets.2024.1376604

**Published:** 2024-07-11

**Authors:** Mariafrancesca Petrucci, Simone de Brot, Daniela Casoni

**Affiliations:** ^1^Faculty of Medicine, Experimental Surgery Facility (ESF), Experimental Animal Center (EAC), University of Bern, Bern, Switzerland; ^2^Department for BioMedical Research, Faculty of Medicine, University of Bern, Bern, Switzerland; ^3^Graduate School for Cellular and Biomedical Science (GCB), University of Bern, Bern, Switzerland; ^4^COMPATH, Institute of Animal Pathology, University of Bern, Bern, Switzerland

**Keywords:** etomidate, propylene glycol, minipigs, side effects, hemolysis, intubation, laryngeal edema, general anesthesia

## Abstract

Etomidate, an agonist of the GABA A receptors, is available for clinical use either in combination with 35% propylene glycol or in a lipid emulsion. Its recognized ability to minimally impact the cardiovascular system made etomidate a suitable option for cardiac-compromised patients. Myoclonus and pain at the injection site are recognized side effects of etomidate in propylene glycol, affecting both human and veterinary species. There is no information available concerning potential side effect in minipigs. In the present case series, we report the side effects related to the use of etomidate in 35% propylene glycol in five Ellegaard Göttingen Minipigs that underwent general anesthesia for cardiac magnetic resonance imaging days or weeks after experimentally induced myocardial infarction. Following intravenous injection of etomidate, laryngeal edema and hyperemia were observed in one case. In another case, tachycardia, apnea, and decreased oxygen saturation, accompanied by laryngeal edema and hyperemia, were observed, which resolved spontaneously in a few minutes. In the arterial or venous samples collected shortly after the induction of general anesthesia, hemolysis was macroscopically visible and subsequently confirmed with a hematological exam in all five cases, as well as hemoglobinuria. Necropsies carried out immediately after euthanasia confirmed macroscopic laryngeal edema, marked diffuse lung alveolar and interstitial edema and hyperemia at histology in one animal, and marked acute lung congestion in another animal. These side effects were not observed when etomidate in a lipid emulsion was injected into another 24 animals. The role played by the different formulations (propylene glycol versus lipidic formulation) has not yet been fully elucidated. Based on our observations, we recommend caution in using the formulation of etomidate in 35% propylene glycol in Göttingen Minipigs.

## 1 Introduction

Minipigs are widely used in biomedical research (1, 2), and most of them undergo cardiovascular surgeries (3–5), which predispose them to cardiovascular adverse events during anesthesia such as arrhythmia and hypotension (3, 6).

Etomidate, R-1-(1-ethylphenyl) imidazole-5-ethyl ester, an agonist of the GABA A receptors, is available for clinical use either in combination with 35% propylene glycol or in lipid emulsion (7). Licensed for human use in 1972, etomidate gained popularity as an anesthesia induction agent due to its minimal effects on the cardiovascular and respiratory systems (8). For this reason, it is recommended for humans and veterinary patients with cardiovascular anomalies (8–13). However, injection of etomidate in 35% propylene glycol has been associated with a number of side effects in both humans and dogs (14–18), including myoclonus, pain at the injection site, and adrenal cortical suppression (7, 18, 19). There have also been rare reports of anaphylactic or anaphylactoid reactions, with the former related to the release of previously sensitized immunoglobulin E (IgE) and the latter unrelated to the release of sensitized IgE (20). The same formulation has also been associated with hemolysis and hemoglobinuria (17, 21, 22).

In the present case series, we report the side effects likely related to the use of etomidate in 35% propylene glycol for anesthesia induction in five Göttingen Minipigs.

## 2 Case series description

The Göttingen Minipigs of this case series were purchased from the official breeder, Ellegaard Göttingen Minipigs (Denmark). These animals were enrolled in a translational study reviewed and approved by the Committee for Animal Experiment of the Canton of Bern, Switzerland (national permission number 33492) and underwent two procedures under two independent general anesthetics: a myocardial infarction (MI) via a closed-chest procedure and a terminal cardiac magnetic resonance imaging (MRI) days or weeks after. For MI induction, intravenous (IV) ketamine (Narketan 10%, vetoquinol) at 1 mg/kg and propofol (propofol 1%, Fresenius Kabi) were selected to induce general anesthesia. For MRI, etomidate in 35% propylene glycol was instead selected due to the expected potential for heart insufficiency. On the day of the MRI, the minipigs were sedated intramuscularly and then transported to the center, where the procedure was carried out. Here, further sedation was administered (if necessary), and blood collection, induction, and maintenance of general anesthesia were carried out. Pulse rate (PR) and SpO_2_ (VM-2500-M, Viamed Limited UK) were monitored until endotracheal intubation. Thereafter, PR, invasive arterial blood pressure, inhaled and exhaled CO_2_ and sevoflurane, heart rate, and rhythm were monitored through a multiparameter monitor (Invivo MR400, MAG Medical equipment). The orotracheal intubation technique has been thoroughly described in the Supplementary material. At the end of the MRI, the minipigs were euthanized under general anesthesia with an IV overdose of pentobarbital (100 mg/kg) (Esconarkon, 300 mg/mL, Streuli Tiergesundheit), with subsequent necropsy performed for tissue collection and analysis.

The minipigs that clinically showed the most severe side effects are presented as cases 1 and 2, and their symptoms are described in detail. The minipigs that presented similar and clinically mild side effects are presented as cases 3, 4, and 5, and the description is cumulated in one single paragraph.

### 2.1 Case 1

A 40 kg, female, 16-month-old minipig underwent terminal MRI 12 days after MI induction. The pre-anesthetic clinical evaluation was unremarkable. A dose of 0.5 mg/kg of midazolam (Dormicum 50 mcg/10 mL, Cito Pharma Services) was injected intramuscularly (IM). Ten min after sedation, pre-oxygenation was started through a face mask at 4 L/min. Since the sedation was insufficient to place a 19-gage (G) port needle on the implanted jugular port-catheter (6 French, Power Port Bard), an additional 0.5 mg/kg midazolam and 2 mg/kg ketamine were injected intramuscularly (IM). Once venous access had been secured, a lactated ringer’s infusion at 5 mL/kg/h was started. General anesthesia was induced with 1 mg/kg etomidate in 35% propylene glycol (Hypnomidate 2 mg/mL, Piramal Critical Care), injected IV as a bolus over 60 s. Thereafter, the depth of anesthesia was deemed adequate for endotracheal intubation (due to the absence of jaw tone and palpebral reflex), and lidocaine 1% was sprayed on the larynx. Endotracheal intubation was subsequently attempted with a 6.5-mm endotracheal tube (ETT) (high volume-low pressure, outer diameter: 8.7 mm, cuff: 21 mm, length: 300 mm) equipped with a stylet. However, when the larynx was visualized, swelling was noticed, and endotracheal intubation failed. At the same time, myoclonus of chewing muscles and tongue was observed. An additional 1 mg/kg etomidate IV was administered to increase the depth of anesthesia and myorelaxation, and apnea occurred. Endotracheal intubation was re-attempted with a smaller (6 mm) ETT after epiglottis displacement but again failed due to the presence of laryngeal edema, accompanied by hyperemic and swallowing laryngeal saccules. Propofol at 0.5 mg/kg and ketamine at 1 mg/kg were subsequently administered IV, and endotracheal intubation was successfully carried out. It required 5 min to secure the airway (from the end of the first etomidate injection to the capnographic confirmation of endotracheal intubation), during which time oxygen was supplemented at a flow rate of 6 L/min. Movement artifacts caused by myoclonus and head manipulation interfered with the SpO_2_ signal from the probe placed on the ear, making it difficult to interpret the values meaningfully. Since the apnea did not resolve spontaneously, mechanical ventilation was started.

**Figure 1:**
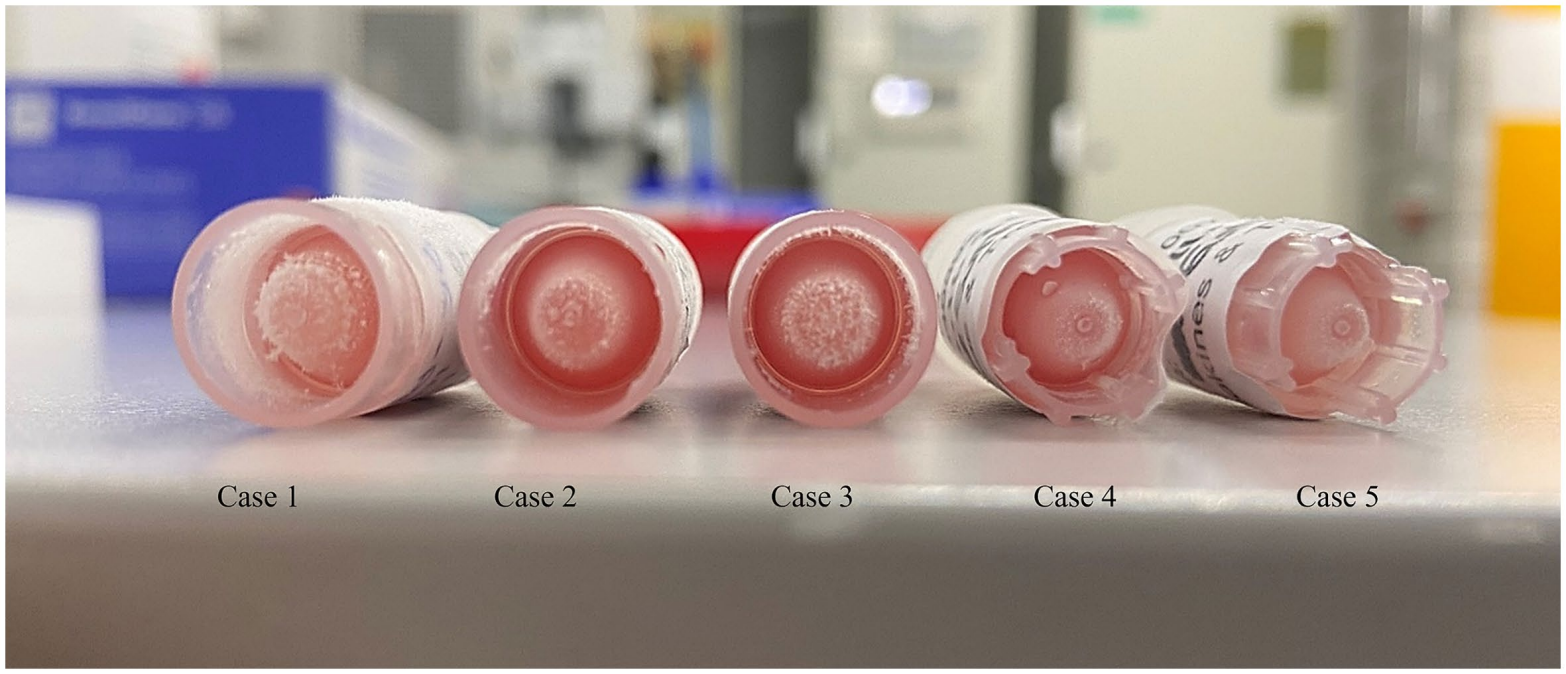
Hemolytic plasma samples (from left to right: sample of cases 1–5).

The presence of hemolysis was macroscopically noticed in the venous blood collected after intubation (Figure 1). Urine was collected post-mortem via cystocentesis. The results of hematology, biochemistry, and urinalysis are detailed in the Supplementary Table 1.

Necropsy confirmed focal extensive MI in the cardiac apex.

### 2.2 Case 2

**Figure 2:**
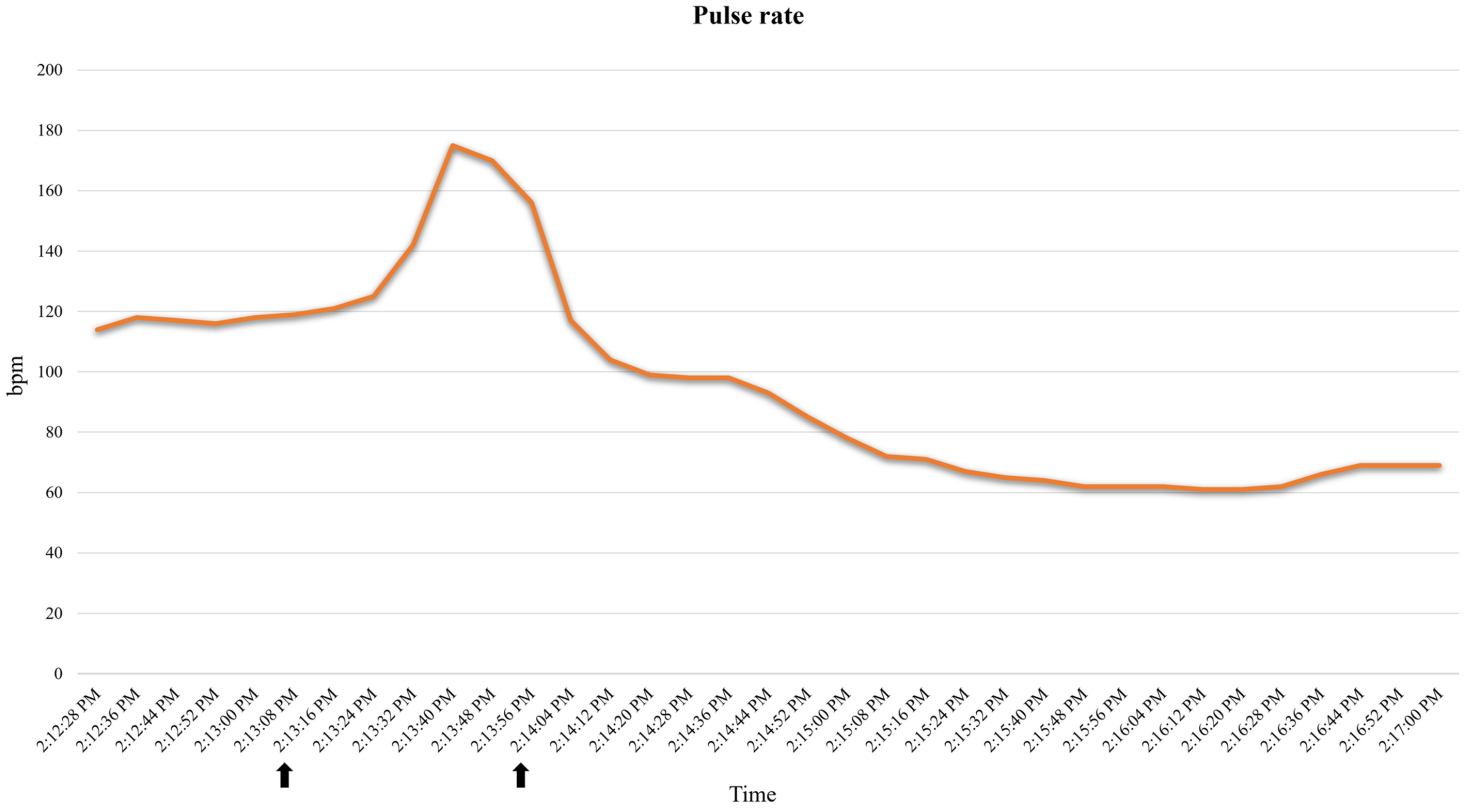
Pulse rate variations recorded by the pulse oximeter in case 2. Increase in pulse rate following etomidate induction. Time span (indicated with arrows): 2:13:16–2:14:04. bpm: beats per minute.

**Figure 3:**
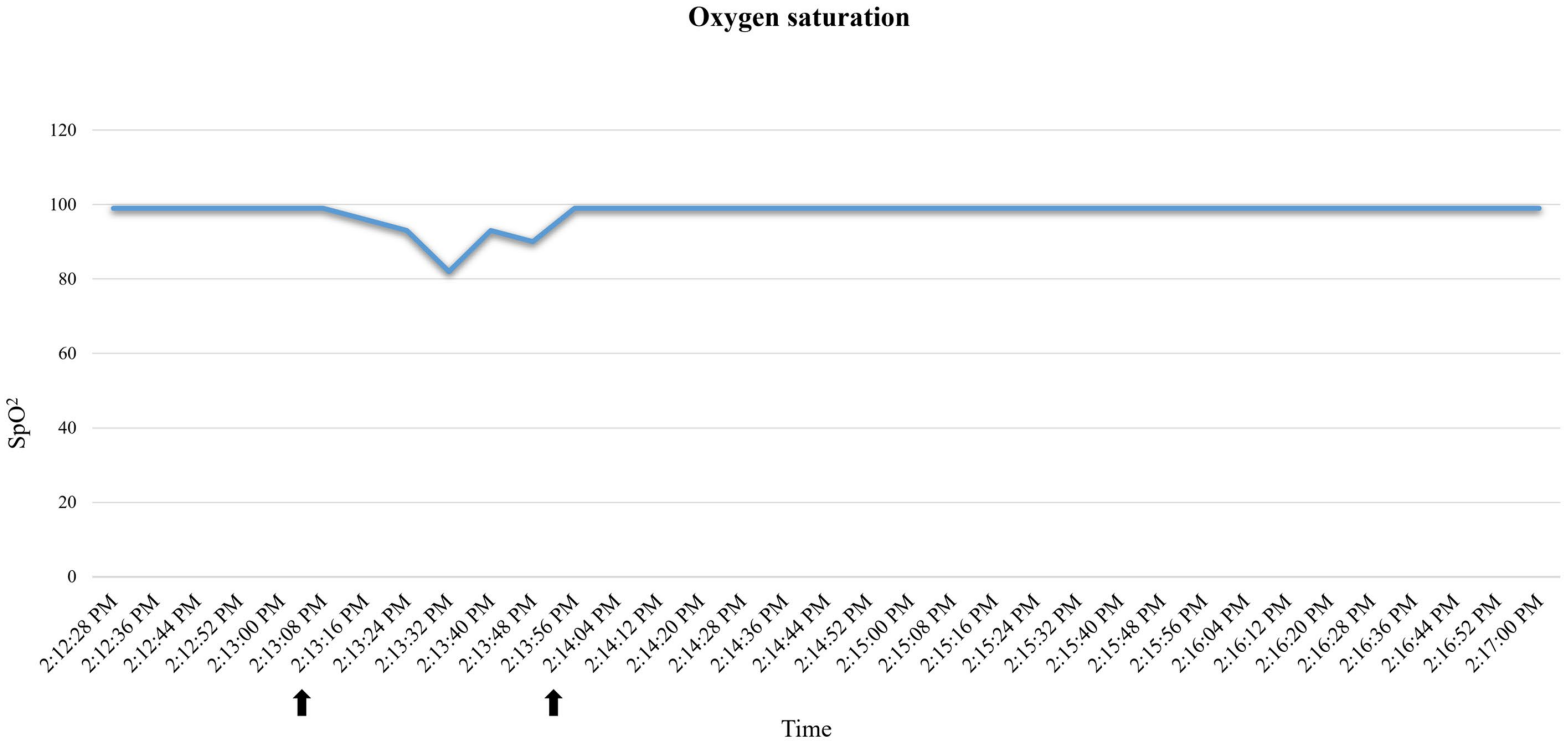
Oxygen saturation (SpO_2_) variations recorded by the pulse oximeter in case 2. Decrease in oxygen saturation after etomidate induction. Reporting as time span the one indicated by the arrows: 2:13:16–2:14:04.

A 35.3 kg, female, 21-month-old minipig underwent terminal MRI 42 days after MI induction. The pre-anesthetic clinical evaluation was unremarkable. Midazolam 1 mg/kg and ketamine 2 mg/kg were injected IM, and 10 min after sedation, pre-oxygenation was started through a face mask (6 L/min). A 22G catheter (0.9*25 mm, BD Venflon Pro Safety) was positioned in the left marginal ear vein, and a 5-ml/kg/h lactated ringers’ infusion was started. Before induction of anesthesia, PR was 111–119 beats per minute (bpm), and a regular breathing pattern (respiratory rate 18–20 breaths per min) was observed. Midazolam at 0.2 mg/kg was administered IV, followed by 2 mg/kg etomidate in propylene glycol, injected as an IV bolus over 60 s. Immediately following its administration, PR suddenly increased to 175 bpm (values reported in Figure 2) and apnea occurred. Endotracheal intubation was immediately attempted. A decrease in oxygen saturation from 99 to 82% was observed (Figure 3). On opening the mouth, the soft palate and epiglottis obstructed direct visualization of the larynx, which was then visualized after three attempts of epiglottis displacement. On visualization of the larynx, hyperemia and edema were observed, particularly in the laryngeal saccules. Lidocaine 1% was sprayed on the larynx, and endotracheal intubation was attempted with a 6-mm ETT, equipped with a stylet. Endotracheal intubation was attempted twice, unsuccessfully. Myoclonus of the muscles of mastication and tongue, as well as increased jaw tone, prevented any further attempt at intubation. Therefore, 1 mg/kg ketamine was injected IV. Afterward, endotracheal intubation was successfully re-attempted despite increased resistance. A mainstream capnograph was connected to the ETT to confirm correct positioning, which gave an end-tidal carbon dioxide (ETCO_2_) value of 65 mmHg. Hypoventilation was confirmed following the connection of the patient to the breathing system (rebreathing system VentStar Anesthesia WT 280 – Bag Cone OD22, Dräger), and mechanical ventilation was started. The airway was secured for 8 min (from the end of the first etomidate injection to capnographic confirmation of endotracheal intubation), during which oxygen was supplemented at a flow rate of 6 L/min. The pulse rate returned to pre-induction values, and SpO_2_ values were restored to >95% 2 min after etomidate administration. Apnea lasted for approximately 1 min, after spontaneous breathing (respiratory rate 7–8 breaths per min) resumed.

The presence of hemolysis was macroscopically noticed in the blood collected from a 22G arterial catheter inserted into the coccygeal artery (Figure 1). Urine was collected post-mortem via cystocentesis and analyzed. The results of hematology, biochemistry, and urinalysis are reported in the Supplementary Table 1.

**Figure 4:**
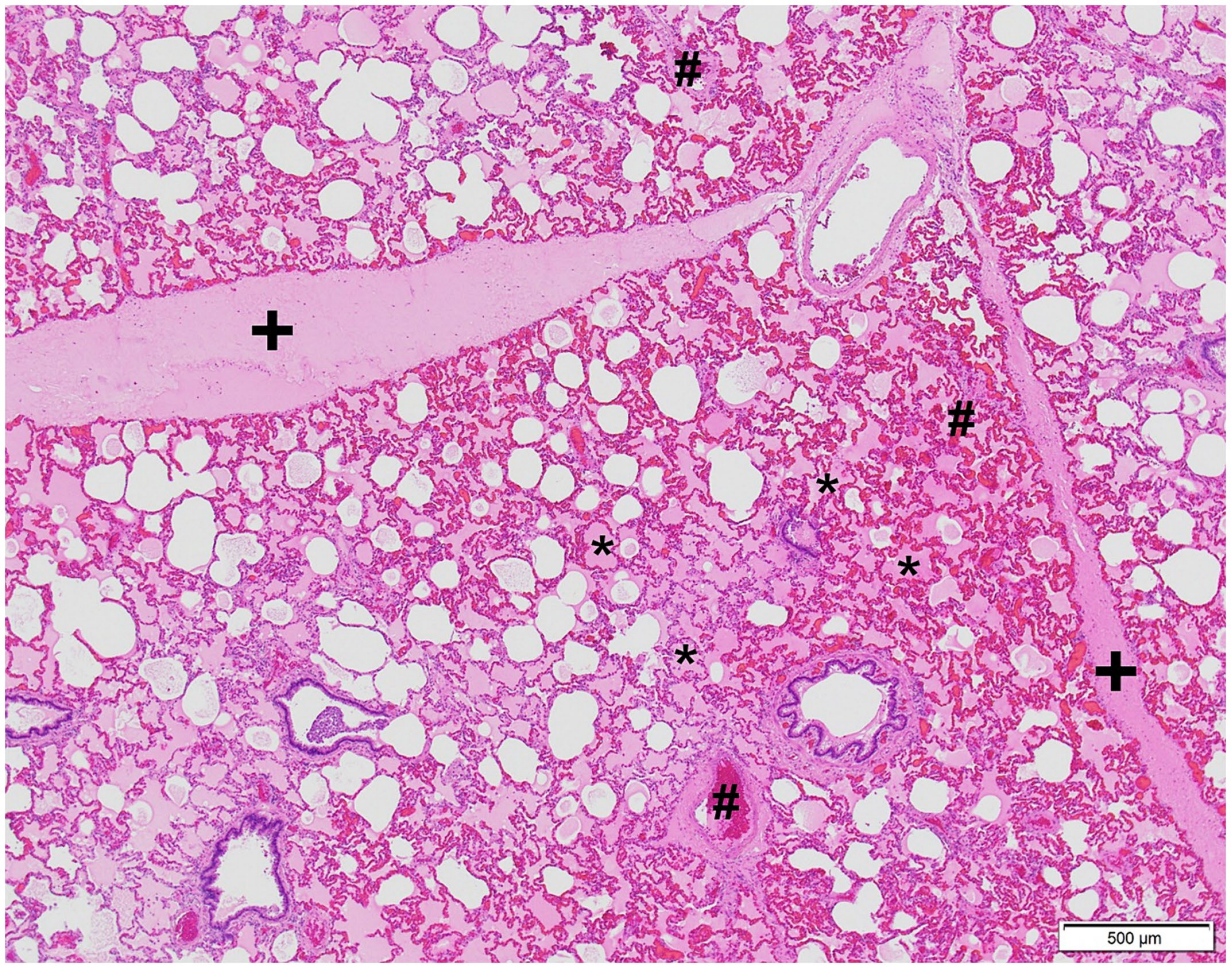
Microphotograph of the lung of case 2. Severe alveolar, interstitial edema, and hyperemia are present. Edema is visible as the extracellular pink substance in alveolar spaces (*), expanding the lung interstitium (+). Hyperemia is characterized by distended and engorged blood vessels (#). Hematoxylin and eosin stain.

In addition to MI, necropsy revealed severe laryngeal edema, marked diffuse alveolar and interstitial edema, and hyperemia of the lungs, which was confirmed histologically (Figure 4).

### 2.3 Cases 3, 4, and 5

Two female and one male minipigs weighing 37.4 ± 3 kg, aged 20 ± 1 months, underwent terminal MRI 38 to 42 days after MI. The pre-anesthetic clinical evaluation was unremarkable, and sedation was carried out as for case 2. An additional 0.2 mg/kg midazolam IM was needed in cases 3 and 4. General anesthesia was induced with midazolam at 0.2 mg/kg followed by etomidate in propylene glycol titrated to achieve the loss of palpebral reflex and jaw tone injected in a 22G catheter in the marginal ear vein (left: case 3 and 5, right: case 4). In case 3, 2 mg/kg with an additional 1 mg/kg etomidate in propylene glycol IV was injected; in case 4, 1.5 mg/kg; in case 5, 1.5 mg/kg with an additional 1 mg/kg. Myoclonus appeared only in case 5 following the etomidate injection. Endotracheal intubation was achieved at the first attempt with a 6.5-mm ETT in all three cases.

A 22G coccygeal arterial catheter was then placed. Blood was collected several minutes following endotracheal intubation, while urine was collected post-mortem via cystocentesis. All collected plasma samples were hemolytic (Figure 1). The hematology, biochemistry, and urinalysis results are reported in the Supplementary Table 1.

Necropsy confirmed the presence of MI in all cases, and a marked acute lung congestion was observed in case 3.

## 3 Discussion

In the present case series, side effects of etomidate in propylene glycol were reported. Hemolysis and hemoglobinuria were present in all five cases, while adverse reactions involving upper and lower airways were observed in two.

Side effects of common anesthesia drug classes such as neuromuscular blocking agents, benzodiazepines, opioids, and induction agents have been reported in humans (23). On the contrary, few reports are present in the veterinary literature (24). In particular, etomidate has only been described to trigger adverse reactions in a low percentage of patients (14–17, 21, 22, 25, 26) and is considered to be the ideal choice for veterinary patients with cardiovascular diseases (9, 11, 13, 27). Indeed, etomidate was selected in these minipigs, expectedly affected by a certain degree of heart insufficiency, due to its reported minimal cardiovascular effects (8, 9, 11, 13). However, etomidate is commercially available in two different solutions: lipidic emulsion or propylene glycol. Propylene glycol is added to etomidate as a stabilizer, emollient, preservative, solvent, and spreader (15). Hypnomidate contains 362.6 mg of propylene glycol in 1 mL, making the drug hyperosmolar (22). This hyperosmolarity has been reported to cause venous pain following an IV injection (25), histamine release, phlebitis, and hemolysis (11, 26). In veterinary medicine, hemolysis has been described in two studies in dogs receiving an etomidate in propylene glycol infusion (17, 22). Moon et al. (17) reported hemolysis and hemoglobinuria in two dogs receiving etomidate infusion (5.9 mg/kg and 15.8 mg/kg total). Moreover, slow recovery time after anesthesia, obtundation, hypothermia, and bradycardia were recorded in one dog. The same has been shown by Ko et al. (22), who described acute hemolysis after the administration of 2 mg/kg followed by a 110 μg/kg/min constant rate infusion of etomidate in propylene glycol for anesthesia induction in dogs. Plasma histamine increase, hypertension and decrease in polymorph count was observed following administration of drugs containing propylene glycol and alcohol in minipigs (28). Information from the manufacturer about hypnomidate in humans reports myoclonus, pain at the injection site, and apnea as commonly observed side effects, while the incidence of hypersensitivity (such as anaphylactic shock, anaphylactic, or anaphylactoid reactions) is unknown. The authors believe this case series is the first report of side effects associated with the use of etomidate in propylene glycol in swine.

The anaphylactic reaction has been defined as a type I hypersensitivity reaction that occurs following exposure to an antigen that stimulates the production of IgE antibodies. If a second exposure to the antigen occurs, this is bound by the IgE antibodies resulting in the release of inflammatory mediators such as histamine, tryptase, prostaglandins, and leukotrienes. In contrast, anaphylactoid reactions are not related to the presence of IgE; therefore, a previous exposure to the antigen is unnecessary (29). However, clinically, they are indistinguishable (23, 29, 30). These reactions involve the cardiovascular, respiratory, and gastrointestinal systems, with the skin and mucous membranes also commonly affected. Clinical manifestations include erythema, edema, pruritus, tachycardia, hypotension, and smooth muscle contractions (23, 30). A clinical severity scale of immediate hypersensitivity reactions was proposed in 1977 (31) and later modified to describe perioperative immediate reactions (23). Four grades have been assigned to these clinical manifestations: in grade I, only cutaneous and mucous signs are present; in grade II, the previous signs are accompanied by tachycardia, hypotension, dyspnea, and gastrointestinal signs; in grade III, all the signs of grade II are present, but they lead to cardiovascular collapse, with cardiac arrest occurring in grade IV (29). According to this grading, the signs in cases 1 and 2 are consistent with a grade II reaction. This reaction resolved spontaneously within a few minutes, with no emergency drugs required. The authors linked the injection of etomidate in propylene glycol to the reported side effects in light of the following considerations. The observed side effects took place within seconds of IV injection of etomidate in propylene glycol, consistent with the temporary lag previously reported for such reactions (30). These minipigs underwent another anesthetic event (for MI induction) before the one described in this case series, in addition to two sedations for procedures related to the study. Etomidate in propylene glycol was not used in any of the previous anesthetic events, whereas midazolam and ketamine had been used multiple times and at consistent dosages, and no comparable complications were observed. Moreover, the individual performing tracheal intubation was involved in all of their anesthetic procedures. Hemolysis and hemoglobinuria were present in all the minipigs that received etomidate as an induction agent, while none of these signs were observed following the previous anesthesia for MI induction. Blood collection, transport, centrifugation, and analysis of the samples were performed consistently, by the same operator and according to a standard operating protocol.

Two of the five minipigs exhibited clinical signs of an anaphylactoid reaction, perhaps due to individual predisposition. Overdosage cannot be claimed, as doses used were similar in all the reported cases and within the suggested dose range commonly used in pigs and other veterinary species (32).

In case 1, a 6.5-mm ETT, which was successfully inserted into the trachea on the first attempt on the day of MI induction, could not be placed after the etomidate injection due to laryngeal edema and had to be replaced with a smaller tube.

Tachycardia and apnea followed by a sudden drop in SpO_2_ observed in case 2 (Figures 3, 4), together with signs of hyperemia and edema of the upper airways, are consistent with an anaphylactoid reaction (29). Unfortunately, arterial blood pressure was not recorded prior to ETT placement; therefore, we could not track the potential hypotension. Tachycardia could also be explained as a sign of nociception, as etomidate injections have been reported to be painful in humans (26). However, the lack of motor response to injection, together with the presence of concurrent apnea and mucous membrane congestion, makes this less likely. Self-limiting apnea can be expected when etomidate is used to induce general anesthesia (33). However, its favorable cardio-respiratory profile has been reported in dogs, and significantly higher PaO_2_ and SaO_2_ have been observed after etomidate induction compared to propofol (27). To the best of the authors’ knowledge, the correlation between the rate of etomidate administration and the incidence of apnea has never been studied. Although rapid IV administration of etomidate may result in apnea, as reported with propofol and alfaxalone (34), the large volume required to achieve the desired dosage for the cases in this series (~20–40 mL was necessary to administer 1–2 mg/kg) precluded rapid IV injection. One could relate the observed tachycardia to hypoxia following apnea. However, considering the general clinical picture, we interpreted tachycardia as a response to a sudden drop in arterial blood pressure, which consequently resulted in a drop in SpO_2_. Indeed: (1) hypoxia was of very short duration to trigger tachycardia, (2) the efficacy of this response depends on the integrity of the respiratory response, the autonomic nervous system, and the vascular system, which was not intact here, and (3) these responses are usually depressed by anesthetic agents (35).

Etomidate in lipid emulsion was administered to the remaining 24 animals included in this translational study, and it is worth mentioning that neither laryngeal edema nor hemolysis was further observed. Although this information has a purely observational nature, it could underline the pivotal role of propylene glycol in triggering these side effects.

Myoclonus is commonly reported following the administration of etomidate in both human and veterinary medicine (7, 18, 27). To reduce its incidence, it has been suggested that etomidate be combined with benzodiazepines or opioids (27, 36). In these minipigs, etomidate was injected only when decreased arousal and myorelaxation were achieved, and further midazolam was administered shortly before induction in all the cases but one. Despite this, myoclonus (primarily involving the muscles of mastication and the tongue) was observed in three cases following the administration of etomidate. The authors consider it unlikely that a higher dose of midazolam could have prevented its occurrence. Myoclonus was further observed in minipigs in which etomidate in a lipid emulsion was administered.

## 4 Concluding remarks

We presented the side effects related to the use of etomidate in propylene glycol in Göttingen Minipigs: difficult intubation related to edema and hyperemia of the laryngeal saccules and tachycardia followed by a drop in SpO_2_ were observed. Hemolysis was also present. The authors recommend caution in selecting etomidate in propylene glycol for its favorable cardiovascular profile in minipigs. Based on our experience, the formulation (propylene glycol versus lipidic emulsion) played an important role in the development of side effects, but this information has not been verified in a controlled prospective trial.

## Data availability statement

The original contributions presented in the study are included in the article/Supplementary material, further inquiries can be directed to the corresponding author.

## Ethics statement

The animal study was approved by the Committee for Animal Experiment of the Canton of Bern, Switzerland (national permission number 33492; cantonal permission number BE 17/2021). The study was conducted in accordance with the local legislation and institutional requirements.

## Author contributions

MP: Conceptualization, Data curation, Investigation, Writing – original draft, Writing – review & editing. SB: Investigation, Writing – original draft, Writing – review & editing. DC: Investigation, Supervision, Writing – original draft, Writing – review & editing, Validation.
