# Supplementary material for: Case report: Side effects of etomidate in propylene glycol in five Göttingen Minipigs
Source: Front Vet Sci. 2024 Jul 11;11:1376604. doi: 10.3389/fvets.2024.1376604 (PMC11270138; doi:10.3389/fvets.2024.1376604)
Supplement: Supplementary file 1 [file Table_1.docx]

Supplementary Material

**Side effects of etomidate in propylene glycol in five Göttingen Minipigs: a case series**

Mariafrancesca Petrucci*, Simone de Brot, Daniela Casoni

* Corresponding Author: mariafrancesca.petrucci@unibe.ch

# Supplementary Table 1

| *Parameters* | *Case 1* | *Case 2* | *Case 3* | *Case 4* | *Case 5* |
| --- | --- | --- | --- | --- | --- |
| BLOOD WORK  (Hematology and biochemistry) | | | | | |
| Plasma macroscopic evaluation | Hemolytic | Hemolytic | Hemolytic | Hemolytic | Hemolytic |
| Hemolysis index | 95 | 146 | 170 | 195 | 232 |
| Total hemoglobin  *g/L* | 115 | 104 | 101 | 97 | 96 |
| Creatinine kinase  *U/L* | 413 | 4685 | 1919 | 1763 | 1870 |
| URINALYSIS | | | | | |
| Urine macroscopic evaluation | Yellow, clear | Yellow/ brown | Yellow, clear | Yellow, clear | Red/brown, clear |
| Protein  *mg/dl* | Negative | 500 | Negative | 75 | 500 |
| Hemoglobin  *Ery/µl* | 250 | 250 | 250 | 250 | 250 |

**Supplementary table 1**. Alteration in blood work parameters and urinalysis in cases 1,2, 3, 4, and 5.

# Orotrachel intubation technique

Minipigs were held in sternal recumbency on a table. The distance between the tip of the nose and the scapular joint was measured to estimate how deep the endotracheal tube should have been introduced. The head of the animal was hyperextended, and a laryngoscope equipped with a Miller blade (KIRCHNER & WILHELM GmbH) was used to visualize the larynx; if the soft palate incarcerated the epiglottis, the base of the tongue was gently pressed downwards in order to displace the epiglottis. Once larynx was visualized, 1.0 ml lidocaine 1% (Lidocain HCl 1%, Bichsel AG) was sprayed and at least 30 seconds were waited before attempting endotracheal intubation. Endotracheal intubation was then performed using an endotracheal tube (high volume-low pressure, Super Safety Clear, Rüsch, Telefrex Medical) equipped with a stylet (Rüsch Flexi-Slip Stylet). The tip of the tube was positioned downward at the level of the glottis and advanced. If resistance was encountered, the tip was retracted, and the tube was rotated 180° about its long axis and gently advanced. Once the endotracheal tube was positioned, the stylet was removed, and correct endotracheal intubation was confirmed using a mainstream capnograph (IRMA CO2 Mainstream Analyser, Viamed). The cuff of the endotracheal tube was then inflated using a 10 ml syringe filled with air until gas stopped audibly escaping during manual breathing bag squeezing with a maximal pressure of 15 cmH_2_O. The endotracheal tube was then connected to a circle rebreathing system (VentStar Anesth WT 280 – Bag Cone OD22).

Orotracheal intubation was carried out in all the minipigs enrolled in the translational trial by the same operator experienced with the species and the procedure, which was supervised during the procedure by a board-certified anesthesiologist.
